# Neuroprotective Potential of Curcumin in Neurodegenerative Diseases: Clinical Insights Into Cellular and Molecular Signaling Pathways

**DOI:** 10.1002/jbt.70369

**Published:** 2025-07-18

**Authors:** Md. Rezaul Islam, Abdur Rauf, Sumiya Akter, Happy Akter, Md. Ibrahim Khalil Al‐Imran, Md. Naeem Hossain Fakir, Gazi Kaifeara Thufa, Md. Tazul Islam, Hassan A. Hemeg, Waleed Al Abdulmonem, Abdullah S. M. Aljohani, Marcello Iriti

**Affiliations:** ^1^ Department of Pharmacy, Faculty of Health and Life Sciences Daffodil International University Dhaka Bangladesh; ^2^ Department of Chemistry University of Swabi Anbar Khyber Pakhtunkhwa Pakistan; ^3^ Padma View College of Nursing Dhaka Bangladesh; ^4^ Department of Clinical Laboratory Sciences, College of Applied Medical Sciences Taibah University Medina Saudi Arabia; ^5^ Department of Pathology, College of Medicine Qassim University Buraydah Saudi Arabia; ^6^ Department of Medical Biosciences, College of Veterinary Medicine Qassim University Buraydah Saudi Arabia; ^7^ Department of Biomedical, Surgical and Dental Sciences University of Milan Milan Italy; ^8^ National Interuniversity Consortium of Materials Science and Technology (INSTM) Firenze Italy

**Keywords:** curcumin, neurodegenerative diseases, neuroprotection, signaling pathways

## Abstract

Progressive neuronal loss and dysfunction characterize neurodegenerative diseases (NDs) such as Alzheimer's, Parkinson's, and Huntington's diseases, spinal cord injury, and stroke, making them difficult to treat. Curcumin, a bioactive substance derived from the turmeric plant (*Curcuma longa*), is interesting due to its potential neuroprotective properties. This review thoroughly shows the cellular and molecular signaling mechanisms that curcumin utilizes to provide neuroprotective effects in NDs. Curcumin regulates several signaling pathways linked to neuroprotection, such as those that reduce oxidative stress, prevent Aβ formation, and decrease neuroinflammation. NF‐κB suppression reduces inflammatory responses, while Nrf2 activation boosts antioxidant response element expression. Furthermore, curcumin enhances autophagy and neurotrophic factor expression, facilitating the removal of harmful protein aggregates. The function of curcumin as a metal chelator is emphasized particularly to iron and other metal dysregulations linked to neurodegenerative processes. Curcumin's capacity to regulate metal ion homeostasis is essential since the pathophysiology of NDs is significantly influenced by metal‐induced oxidative stress and toxic buildup. It shows potential therapeutic effects by reducing oxidative damage and chelating excess metals. Clinical research indicates that curcumin can penetrate the blood‐brain barrier, making it an effective treatment option. The regulation of these pathways reduces neuronal damage and improves neurons' survival and functionality. In addition, curcumin's anti‐inflammatory properties and low toxicity make it a promising long‐term treatment option for NDs. Therefore, this review emphasizes the potential of curcumin as a targeted neuroprotective compound, presenting recent clinical insights and experimental data. Future studies should optimize curcumin formulations and delivery systems to enhance its bioavailability and therapeutic efficacy.

## Graphical abstract

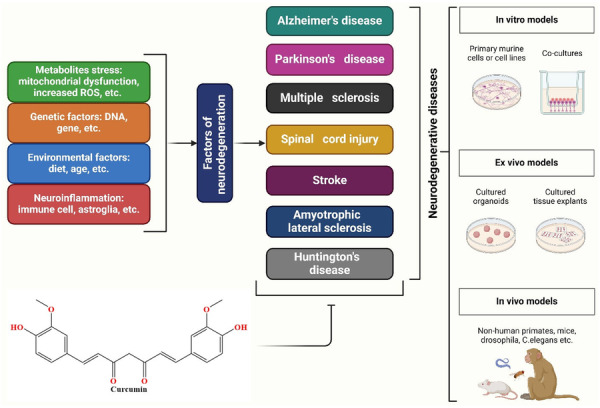

## 1 Introduction

**Figure 1:**
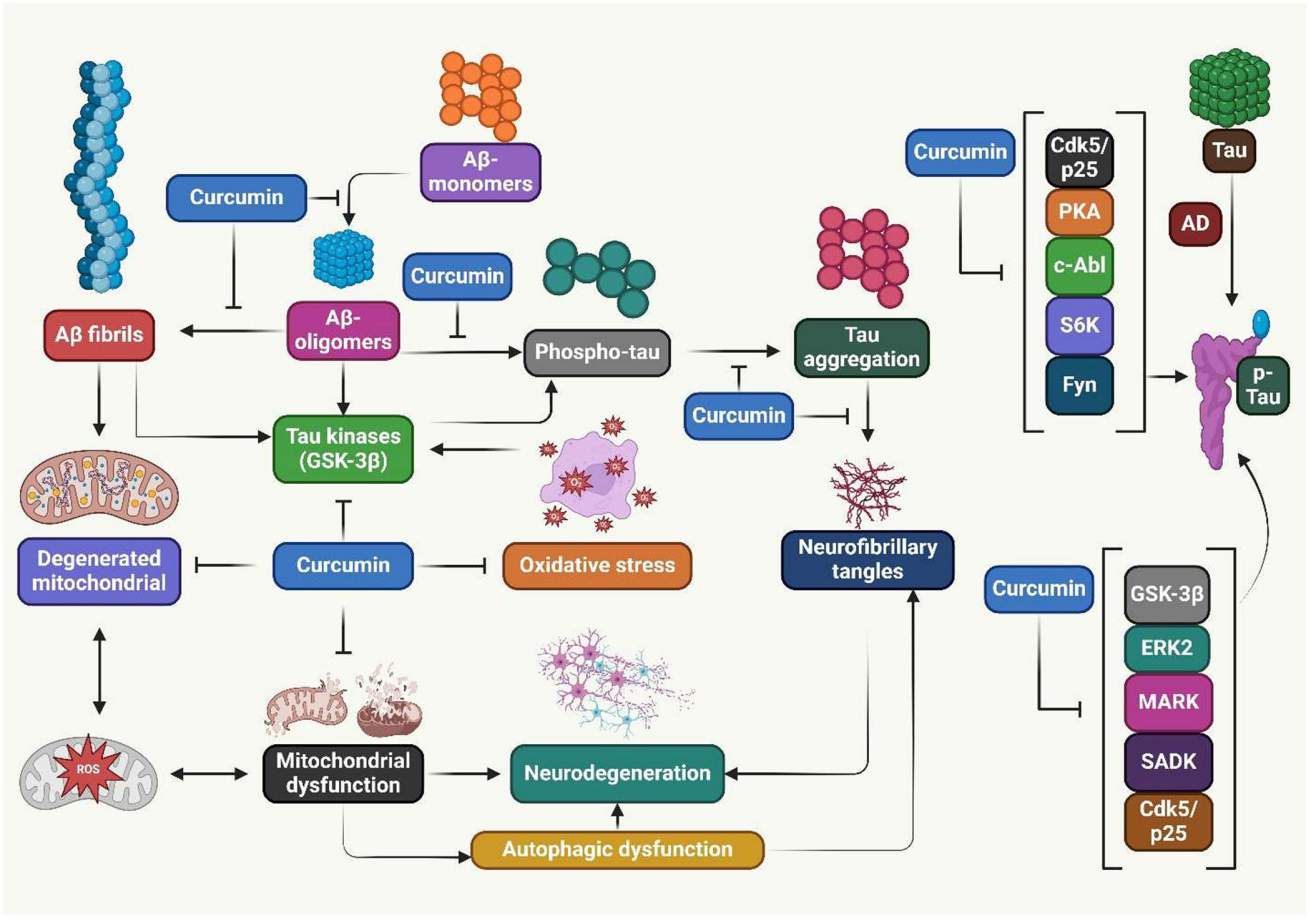
Curcumin activated microglia, promoted Aβ breakdown through phagocytosis, neutralized Aβ, prevented Aβ from entering the brain, and increased Aβ out of the brain into the bloodstream.

The brain is a highly metabolically active organ that necessitates significant oxygen for proper functioning. Brain cells have mitochondria where oxidation–reduction events occur in the presence of oxygen to produce ATP. Elevated concentrations of free radicals in the central nervous system (CNS) significantly influence the pathophysiology and progression of neurodegenerative and inflammatory processes [1]. The function and structure of the CNS's neuron population gradually deteriorate in conditions known as neurodegenerative diseases (NDs) [2]. NDs such as Alzheimer's disease (AD), Parkinson's disease (PD), Huntington's disease (HD), Multiple sclerosis (MS), dementia with Lewy bodies (DLB), progressive supranuclear palsy (PSP), corticobasal degeneration, and prion disease pose a major risk to human health [3, 4, 5]. The current number of Americans over 65 with AD is 6.7 million, with over a million having PD, and over 200,000 having or at risk of developing HD [6]. By the middle of the century, there will be 152 million dementia patients globally, with low‐ and middle‐income nations expected to see the most increase in cases. By 2050, there could be a significant rise in the number of AD patients (≥ 65 years) in America, from 5.8 million to 13.8 million [7]. MS is prevalent in Asia and Oceania at 37.89/100,000, with an incidence of 2.40 per 100,000 [8]. Traumatic spinal cord injury (TSCI) affects 299,000 people in the United States at present; the yearly incidence of TSCI cases per million individuals is 54, or roughly 18,000 new cases annually [9, 10, 11]. Furthermore, non‐traumatic SCI (NTSCI) prevalence estimates from Canada show 1120 cases per million people [12]; extrapolating this number to the US would result in an additional 372,000 cases of NTSCI. The incidence of non‐TSCI is higher than that of TSCI in several nations, and it is expected to go up further as the population ages [13, 14]. ALS is a severe ND causing the gradual loss of voluntary muscles. With a prevalence of 6–9 per 100,000 people, an incidence of about 2 per 100,000 person‐year, and a lifetime risk of almost 1 in 350, ALS affects people worldwide [15, 16, 17]. Numerous natural alterations that cause neuronal malfunction and death are the hallmarks of NDs. Apoptosis, oxidative and nitrosative stress, mitochondrial malfunction, and uncontrollably high levels of neural inflammation are the results of these alterations. Mitochondrial DNA mutations, mitochondrial transport chain function anomalies, and insufficient ATP generation regulation due to excess free radicals damage proteins. Toxic byproducts including reactive nitrogen species (RNS), free radicals, and reactive oxygen species (ROS) are produced during aerobic mitochondrial metabolism [18]. Moreover, external contaminants like smoke and radiation as well as genetic diseases raise the levels of ROS. ROS interact with proteins, enzymes, lipids, DNA, and RNA, facilitating electron reception and donation, and influencing their structures and activities. The synergistic action of several antioxidants deactivates or stabilizes free radicals, protecting cells. Antioxidants are organic substances in the body or external compounds consumed through the diet that bind redox metals and scavenge free radicals [19]. Research is focusing on discovering plant items as potential therapeutic agents for developing new medications with disease‐protective properties. Natural products with anti‐inflammatory properties may serve as effective foundations for effective treatment plans. Natural plant‐based medications and synthetic alternatives are used for treating AD by inhibiting the CNS [20]. Curcumin [1,7‐bis(4‐hydroxy‐3‐methoxyphenyl) ‐1,6‐heptadiene‐3,5‐dione], a pharmacologically active polyphenol from *Curcumin longa*, has various biological properties including anti‐inflammatory and antioxidant [21]. Curcumin's therapeutic potential is limited by the Blood‐Brain Barrier (BBB), despite its potential to improve NDs such as MS, PD, prion disease, stroke, anxiety, depression, and aging. It has therapeutic efficacy and innovative strategies to overcome its low bioavailability across the BBB. Curcumin delivery across the BBB has been accelerated using various approaches such as liposomes, micelles, polymeric nanoparticles, exosomes, and dual‐targeting nanoparticles [22]. Moreover, curcumin has been proposed as a potential treatment for several NDs (Figure 1). It can alter several signaling pathways such as Nrf2 and NF‐κB, which are implicated in the onset of NDs [23, 24]. Additionally, curcumin has shown therapeutic benefits against NDs and broad‐spectrum neurotoxic substances [25]. Furthermore, curcumin has strong anti‐inflammatory and antioxidant properties and has shown potential in treating AD and PD. Preclinical data supports its potential to affect multiple molecular targets, fight inflammation and oxidative damage, and its neuroprotective properties [26]. In addition, curcumin's inexpensive and nontoxic nature has led to interest in nutraceutical treatments for NDs. Its potential health benefits may be due to its ability to prevent aging‐related protein insolubility and aggregation, which is linked to numerous age‐related diseases [27]. Moreover, curcumin binds to amyloid β‐sheet conformations, scavenges free radicals, and restores inflammatory homeostasis [28]. Chemical imbalances in the concentration of metal ions, particularly copper and iron, can promote the aggregation of proteins and peptides, which in turn can cause oxidative damage. Therefore, metal chelators play a crucial role in treating NDs. Curcumin, known as the “golden spice,” has antioxidant and anti‐inflammatory properties and is known for its ability to chelate metal ions through its keto‐enolic moiety. Curcumin sequesters metal ions to limit misfolded protein deposition, reduce Fenton and Haber‐Weiss processes, and bind ligand molecule's neuroprotective properties. This study showed curcumin‐based chelators' role in neuroprotection and the development of therapeutic medicines for NDs [29]. Transition metals are crucial for pathophysiological processes in common NDs, and daily exposure to metals can disrupt homeostasis. Flavonoids and curcuminoids, known for their antioxidant and neuroprotective properties, can develop effective treatment plans. Fisetin and curcumin can either inhibit or accelerate the neurodegenerative process [30]. NDs such as AD, PD, and HD are linked to neuroinflammation. Cytokines suppressive anti‐inflammatory drugs like curcumin may reduce neuroinflammation and improve clinical outcomes. Curcumin affects transcription factors and pro‐inflammatory pathways, but its in vivo bioavailability is limited. High bioavailability curcumin formulations are being developed to increase therapeutic concentrations [31]. A study using rats with cerebral contusion injuries showed curcumin's neuroprotective effects. The rats were given either vehicle or curcumin, and the size of brain lesions was reduced. The treatment with curcumin significantly improved traumatic brain injury (TBI) in rats [32]. This review demonstrates the neuroprotective benefits of curcumin in NDs by reducing OS, inflammation, and neuronal death. Moreover, clinical research shows it reduces symptoms and enhances cognitive function.

## 2 Curcumin: Structure and Chemistry

**Figure 2:**
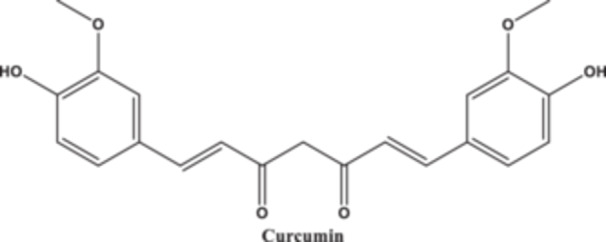
It illustrates the chemical structure of curcumin.

Curcumin (Figure 2), also called diferuloyl methane, is a symmetric molecule. It is known by its IUPAC designation, (1E,6E)−1,7‐bis(4‐hydroxy‐3‐methoxyphenyl). The molecular weight of −1,6‐heptadiene‐3,5‐dione is 368.38, and its chemical formula is C_21_H_20_O_6_. Two aromatic ring systems with o‐methoxy phenolic groups are joined by a seven‐carbon linker made up of an α,β‐unsaturated β‐diketone moiety, making up its three chemical entities [33, 34, 35]. Curcumin is a diferuloyl methane molecule that has two ferulic acid residues connected by a methylene bridge. An aromatic o‐methoxy phenolic group, α, β‐unsaturated β‐diketo moiety, and a seven‐carbon linker are its three main functions. Curcumin's biological activity is attributed to its chemical structural characteristics, which include its ability to donate electrons or hydrogen to ROS. Curcumin interacts with various biomolecules through covalent and noncovalent binding. Curcumin's noncovalent interactions are caused by its hydrophobicity and hydrogen bonding, which result from its tautomeric and aromatic structures and the flexibility of the linker group. The Michael reaction occurs when the α, β‐unsaturated β‐diketone moiety covalently binds with protein thiols. Some metal complexes show enhanced antioxidant activity as enzyme mimics, and the β‐diketo group reduces metal‐induced toxicity by forming chelates with transition metals. Curcumin's particular functional groups are being modified to form new analogues with enhanced action [36]. Additionally, a study demonstrated two potentially beneficial derivatives of curcumin, dicarbomethoxycurcumin, and dicarboethoxycurcumin, by treating it with methyl‐ or ethylchloroformate in the presence of potassium hydroxide. These two derivatives are highly beneficial in curcumin chemistry due to their high purity [37]. Furthermore, curcumin, a stable crystalline form in acidic and neutral pH environments, has two novel metastable enol polymorphs. The study found that solubility and hardness are inversely related, with a softer polymorph being more soluble [38].

## 3 Curcumin: Bioavailability and Metabolism

Curcumin is found in the widely used South Asian spice turmeric. It is used as a food coloring and additive due to its bright yellow color. It also has various tautomeric forms, with the enol form being more stable in solution and the solid phase being the most stable [39]. Additionally, curcumin has health benefits but is limited in food due to its low solubility and bioavailability [40]. Water does not dissolve the hydrophobic chemical curcumin. It dissolves readily in ethanol, methanol, acetonitrile, and ethyl acetate but has poor solubility in hydrocarbon solvents [35]. Curcumin can exist in tautomeric keto‐enol conformations, and the pH, solvent polarity, and temperature all affect how concentrated the different keto‐enol forms are. It, found in keto form, is more stable in basic conditions (pH > 8) than in neutral and acidic conditions (pH 3–7) [35, 36]. This compound's aromatic groups give curcumin its hydrophobicity and low solubility in water. The activity of curcumin is dependent on the aromatic o‐methoxyphenol, the α,β‐unsaturated β‐diketo moiety, and seven carbon linkers. Curcumin's antioxidant activity is caused by a reaction between o‐methoxyphenol and methylenic hydrogens, which donate hydrogen atoms to ROS. It forms both covalent and noncovalent bonds with a variety of biomolecules to interact with them. Proteins' −OH, −SH, and −SeH groups are covalently bound by the β‐diketone moiety; also, the β‐diketone groups form chelates with heavy transition metals (Cu^2+^, Zn^2+^, and Fe^3+^) that lessen their toxicity [41]. Several antioxidant enzymes, including heme oxygenase‐1, glutathione peroxidase, catalase, SOD, and NADPH oxidase, are also made more active by curcumin [42, 43]. Curcumin's low solubility, limited BBB permeability, and liver degradation make it challenging to deliver. Various nanocarriers have been developed for its delivery [44, 45, 46]. In addition, curcumin derivatives are converted into curcumin, dihydrocurcumin, tetrahydrocurcumin, hexahydrocurcumin, and octahydrocurcumin glucuronide derivatives in the presence of UDP glucuronosyltransferase. Additionally, curcumin reacts with the enzyme phenol sulfotransferase to develop curcumin sulfate. Curcumin injected intraperitoneally experiences reduction processes [41]. Recent developments in delivery technologies may increase curcumin's oral bioavailability, overcoming its limited oral delivery due to low solubility, bioaccessibility, and rapid degradation [47]. The poor pharmacokinetic profile of curcumin inhibits its numerous health benefits. Its low absorption and quick excretion make it difficult to treat. Researchers have tested strategies like suppressing metabolism and using innovative oral administration systems to combat this [48].

## 4 Curcumin in Neurogenerative Diseases

The use of curcumin for the prevention and treatment of neurodegenerative diseases is reported in Table 1.

**Table 1:** Curcumin is used to prevent and treat NDs.

Neurodegenerative diseases	Study model	Dose/conc.	Findings	References
Alzheimer's disease	SH‐SY5Y neuroblastoma cells	0–20 or 5 μM	Effect on generating Aβ in neuroblastoma cells and the in vitro expression of PS1 and GSK‐3β	[49]
	Tg2576 APPsw mice	—	Reduced amyloid plaque burden, insoluble Aβ peptide, and carbonyls	[50]
	Mice	25 mg/kg	Protected human neuronal cells from oxidative damage	[51]
	Tg2576 mice	—	Improved the bioavailability in treating AD	[52]
	Rats	300 mg/kg	Inhibited amyloid sedimentation and accelerates the disaggregation of amyloid plaque	[53]
	SHSY5Y cells	—	Prevented Aβ‐induced mitochondrial and synaptic toxicities in AD	[54]
	Male adult Wistar albino rats	100 mg/kg	Reduced neurotoxicity in a specific animal model of AD	[55]
	3x‐Tg‐AD mice	150 mg/kg	Improved behavior, inflammation, and Aβ accumulation in a mouse model of AD	[56]
	B6C3‐Tg mice	200 mg/kg	Improved cognitive function and reduced soluble Aβ‐peptide production	[57]
	APP/PS1 mice	50 and 200 mg/kg	Improved spatial learning and memory abilities and reduced amyloid plaque burden in the hippocampus	[58]
Parkinson's disease	Male Sprague–Dawley rats	40 mg/kg	Inhibited α‐synuclein aggregation in a lipopolysaccharide‐induced PD model	[59]
	Male C57BL/6 mice	50 mg/kg	Mitigated the effects of glutathione depletion in PD	[60]
	SH‐SY5Y cells	—	Decreased α‐synuclein‐induced cytotoxicity in a cell model of PD	[61]
	Male albino rats	30 mg/kg	Decreased neurotoxic effects, degenerative histological changes, and OS in a PD rat model's cerebellar cortex	[62]
	Male Sprague–Dawley rats	5, 10, and 20 mg kg^−1^	Increased monoaminergic neurotransmitters and upregulates BDNF, TrkB, and PI3K protein expressions	[63]
	Male Swiss mice	25 and 50 mg/kg	Improved motor impairment, decreased lipoperoxidation, modified antioxidant defenses, and protected inhibition of complex I	[64]
	A53T α‐synuclein cells	10 mM	Reduced A53T α‐synuclein accumulation by downregulating mTOR/p70S6K signaling	[65]
	Male C57BL/6 mice	50 mg/kg	Inhibited JNKs and prevented dopaminergic neuronal loss in a mouse model of PD	[66]
	Swiss male albino mice	80 mg/kg	Inhibited monoamine oxidase‐B by curcumin in a mouse model of PD induced	[67]
	Male Wistar rats	10 and 30 mg/kg	Prevented curcumin‐induced dopaminergic neurotoxicity and heavy metal‐related Parkinsonism	[68]
Multiple sclerosis	Female Lewis rats	—	Reduced neurological symptoms in the MS EAE model by regulating inflammation and OS, enhancing neuroprotection and myelin repair	[69]
	Female C57BL/6 mice	100 mg/kg	Reduced neuroimmune imbalance and demyelination in MS	[70]
	Male C57BL/6 mice	100 mg/kg	Decreased testicular damage in a mouse model of MS	[71]
	Female C57BL/6 mice	20 mg/kg	Decreased pro‐inflammatory cytokine levels, and enhanced anti‐inflammatory cytokine expression	[72]
	Female Lewis rats	100 and 200 mg/kg	Improved EAE by inhibiting the production of IL‐17	[73]
	Mice	—	Reduced EAE severity and increased TGF‐β and IL‐10 levels but decreased IL‐1, IFN‐γ, and IL‐17 expression	[74]
	Wistar rats	100 mg/kg	Reduced OS in MS in rats	[75]
	Mice	100 mg/kg	Regulated anti‐inflammatory responses through the AXL/JAK2/STAT3 signaling pathway in EAE	[76]
	Female SJL/J mice	50 or 100 μg	Inhibited EAE by inhibiting IL‐12 signaling in T lymphocytes via the Janus Kinase‐STAT pathway	[77]
	T cells	20 μg/mL	Inhibited inflammatory cytokine production by human monocytes and MS and EAE in animals	[78]
Spinal cord injury	Male Sprague–Dawley rats	40 mg/kg	Enhanced motor function recovery and decreased spinal cord edema in a rat acute SCI model	[79]
	Male Sprague–Dawley rats	100 mg/kg	Improved the outcome of SCI by reducing the inflammatory response	[80]
	Female Sprague–Dawley rats	60 mg/kg	Improved SCI recovery in experimental animals	[81]
	Male Wistar rats	200 mg/kg	Improved early functional outcomes after experimental SCI	[82]
	Male Sprague–Dawley rats	100 mg/kg	Improved outcomes by decreasing the TLR4/NF‐κB inflammatory signaling pathway in the SCI	[83]
	Female KM mice	50, 100, and 200 mg/kg	Effect on acute SCI in mice by inhibiting inflammation and regulating the TAK1 pathway	[84]
	Female Sprague–Dawley rats	60 mg/mL/kg	Activated NSC proliferation in vitro and combination with stem cell therapy, induced profound recovery from severe SCI	[85]
	Sprague–Dawley rats	200 mg/kg	Reduced inflammation, and astrogliosis, and improved functional recovery and histologic outcomes in SCI	[86]
	Male Wistar rats	—	Improved functional outcomes by modifying pro‐inflammatory cytokines and reducing NF‐κB activity, thus having therapeutic potential for early SCI treatment	[87]
	Rats	200 mg/kg	Promoted oxidative balance and motor function recovery in rats after traumatic SCI	[88]
Stroke	Male C57BL/6 mice	150 mg/kg	Prevented ischemic stroke by titrating microglia and macrophage polarization	[89]
	Male Sprague–Dawley rats	300 mg/kg	Improved the healing process of ischemic stroke in rats by preserving the integrity of the BBB	[90]
	Male Sprague–Dawley rats	300 mg/kg	Prevented focal cerebral ischemia‐reperfusion injury and stimulated neurogenesis by activating the Notch signaling pathway	[91]
	Male Wistar rats	300 mg/kg	Protected reperfusion injury in rats after ischemic stroke by inhibiting the expression of NF‐κB, ICAM‐1, MMP‐9, and caspase‐3	[92]
	Male C57BL/6 mice	100, 200, 300, 400 mg/kg	Prevented neural cells in N2a cells and the mouse brain from ischemic stroke	[93]
	Male C57BL/6 J mice	150 mg/kg	Reduces white matter injury after ischemic stroke by blocking microglia/macrophage pyroptosis	[94]
	Wistar rats	30 mg/kg	Improved behavioral recovery, reduced hematoma size, decreased weight loss, and modulated antioxidant responses	[95]
	Female Wistar Hannover rats	300 mg/kg	Reduced neurological scores and apoptotic index in the ischemic group compared to the curcumin‐treated group	[96]
	Male Sprague–Dawley rats	300 mg/kg	Upregulated Prdx6 protein expression and decreased OS causing neuroprotective effects against ischemic damage	[97]
	Male C57BL/6 mice	100 mg/kg	Promoted anti‐inflammatory microglia polarization via the Nrf2 pathway, decreased ROS, oxidative mediators, and pro‐inflammatory cytokines	[98]
Amyotrophic lateral sclerosis	Transgenic mice	—	Evaluated GT863's inhibitory effect on SOD1 aggregation, antioxidant, anti‐inflammatory, and neuroprotective effects	[99]
	NSC‐34 cells	—	Improved mitochondrial dysfunction in mutated TDP‐43 cells	[100]
Huntington's disease	R6/2 transgenic mice	25 mg/kg	Prevented neuropathology and GI dysfunction, and preserve intestinal contractility, potentially improving debilitating symptoms associated with HD	[101]
	Wistar rats	40 mg/kg	Reduced neurochemical and behavioral deficits in an experimental model of HD	[102]
	CAG140 KI mice	—	Improved neuropathology and transcriptional impairments in CAG 140 knock‐in mice, indicating its potential beneficial effect on HD	[103]
	Drosophila	10 μM	Improved locomotion ability and eclosion behavior in HD flies	[104]
	Drosophila	10 μM	Decreased higher crystal cell count and phenoloxidase activity in HD flies, improved phagocytic activity, and ROS levels	[105]
	Wistar rats	25 and 50 mg/kg	Demonstrated strong antioxidant and protective effects against QA‐induced behavior in rats	[106]

### 4.1 Alzheimer's Disease

**Figure 3:**
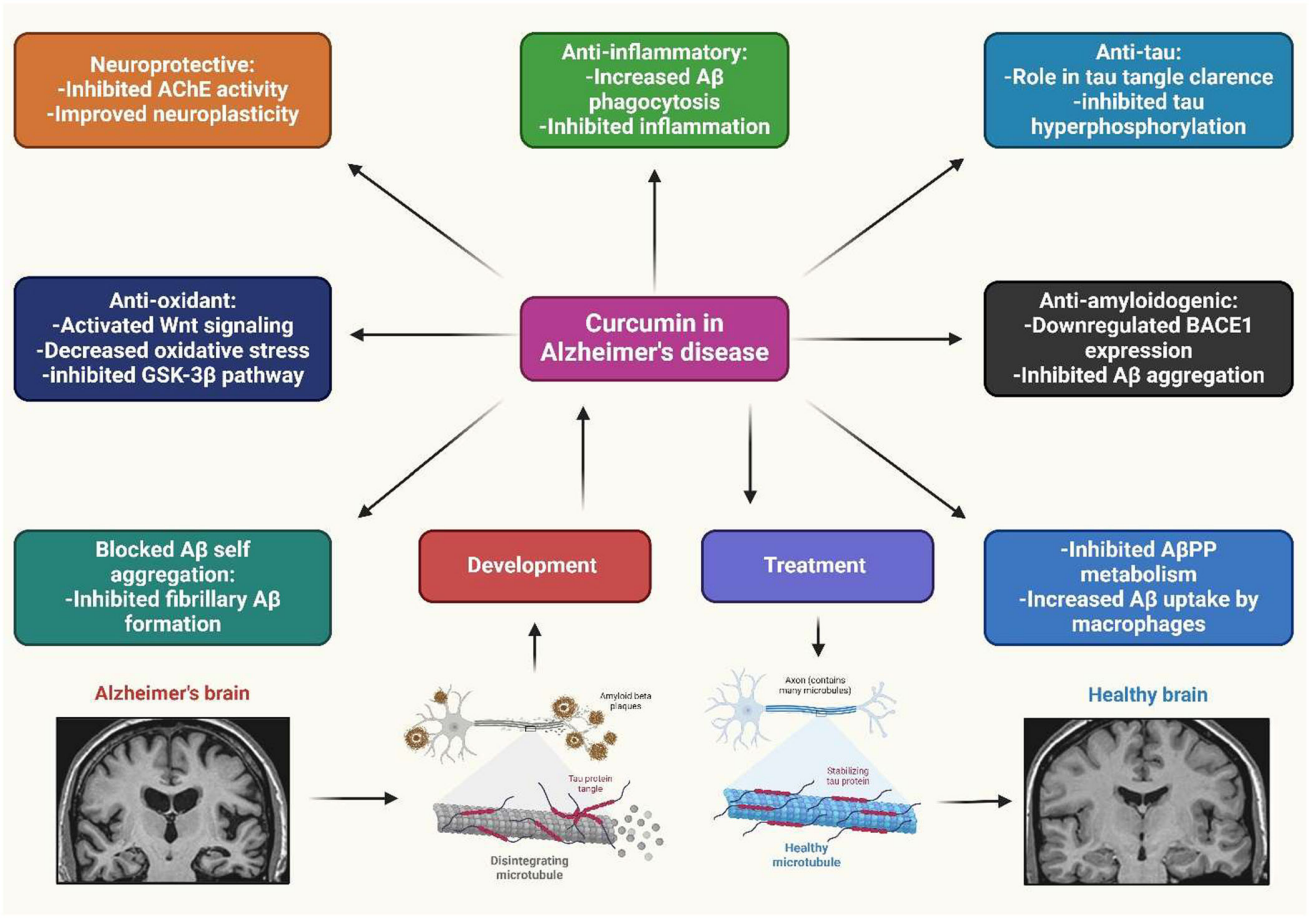
Curcumin demonstrated the neuroprotective properties. It prevented and treated AD.

AD is an ND caused by Aβ plaque deposition and neuroinflammation. It is caused by microglial cells, ROS, NF‐κB, and apolipoprotein E. Current FDA‐approved medications treat symptoms, but curcumin is explored for potential therapeutic agents [107]. AD is a prevalent type of dementia, with modern medicine offering few options due to expensive, side‐effect‐prone medications. Curcumin has the potential to treat AD [108]. A study showed curcumin in AD diagnosis, prevention, and treatment. Curcumin's strong affinity for Aβ and its natural fluorescent nature make it an early diagnostic probe. It has preventative benefits for chronic diseases like hypertension, hyperlipidemia, and cerebrovascular disease. Its anti‐inflammatory, antioxidant, and metal iron chelating properties support its use in AD treatment and prevention [109]. Curcumin may have therapeutic potential in AD pathogenesis. Oral curcumin therapy improved behavioral impairment in animal models of AD by inhibiting tau phosphorylation and Aβ deposition. However, more study is required to determine curcumin's clinical utility in AD prevention and treatment (Figure 3) [110]. Additionally, curcumin enhances cognitive performance in AD patients. Research suggests that curcumin's benefits, including decreased Aβ plaques, and delayed neuronal degradation, may help manage and prevent AD [111]. Furthermore, curcumin treats AD by preventing Aβ plaque development, reducing tau hyperphosphorylation, binding to copper, lowering cholesterol, altering microglial activity, blocking acetylcholinesterase, and acting as an antioxidant [112]. In addition, curcumin has potential neuroprotective effects. However, its hydrophobic nature increases the risk of brain buildup and BBB penetration [113]. However, curcumin reduces amyloid pathology in animal models of AD. However, there is limited evidence on its safety, tolerability, and bioavailability in older adults, prompting a study on curcumin in AD patients [114]. Curcumin has cognitive‐improving and neuroprotective properties, potentially helping to postpone or prevent AD. However, curcumin's low solubility and bioavailability and its selection of patients with significant neuropathology have limited its clinical trials. However, there is new hope for curcumin‐based therapy, particularly in treating early AD pathology and developing novel curcumin formulations to boost bioavailability [115]. Additionally, curcumin is gaining interest in potential AD therapies. It reduces Aβ toxicity and inhibits AD‐related pathways [116]. In addition, curcumin triggers the activation of cytoprotective proteins in the phase II response. It may target AD pathways, but its insolubility in water and poor bioavailability inhibit clinical trials, necessitating its combination with other medications or novel delivery methods [117]. Furthermore, curcumin showed potential anti‐amyloidogenic properties. Both showed similar brain levels, but curcumin effectively reduced amyloid plaque, carbonyls, and insoluble Aβ peptide burden. Tetrahydrocurcumin decreased phospho‐c‐Jun NH2‐terminal kinase and Tris‐buffered saline‐soluble Aβ, but not plaques or insoluble Aβ. Curcumin inhibited Aβ aggregation. It has a dienone bridge to decrease plaque development and protein oxidation in AD [50]. Another study investigated the effects of curcumin and its modified formulations on AD models in both experimental and human settings due to its limited oral bioavailability [118]. A study showed that curcumin against Aβ‐induced damage in AD. It reduces Aβ aggregation, passes the BBB, and protects neurons from aging and Aβ. It also improves synaptic functioning and mitigates cognitive decline in AD mice models [119]. Curcumin reduces amyloid levels and prevents cognitive deficits in animal models of AD. Its antioxidant and anti‐inflammatory properties may be due to metal chelation, which can mitigate oxidative neurotoxicity and amyloid formation. It also protects against Aβ toxicity and reduces inflammatory damage [120]. Another study investigates the protective mechanisms of curcumin against Aβ in AD neurons. Aβ inhibits mitochondrial biogenesis, reduces synaptic activity, and impairs mitochondrial dynamics. It boosted synaptic protein biogenesis, decreased fission machinery, and increased mitochondrial fusion activity. It also is more effective in preventing AD‐like neurons than in treating them, making it a viable medication for AD treatment [54]. Curcumin reduces brain inflammation and protects against Aβ‐induced neurotoxicity. It suppresses Aβ synthesis and aggregation in AD. Strategies are being developed to enhance delivery methods or produce analogs that mimic curcumin's neuroprotective properties [121]. In addition, curcumin derivatives with keto‐enol tautomerism exhibit high binding activity to Aβ fibrils/aggregates, but not for Aβ monomers. It is crucial for binding to Aβ aggregates, and their keto‐enol tautomerism could be a unique target for developing amyloid‐binding drugs for AD detection and therapy [122]. Moreover, curcumin restores neuroinflammatory networks linked to AD by lowering CD33 and increasing TREM2 expression. Curcumin at low and high doses promoted the recruitment of microglia to amyloid plaques and their phagocytosis [123]. Curcumin treats AD through its role in neurogenesis, decreased pro‐inflammatory cytokines, and deactivated GSK‐3β. It also reduced plaque formation and Aβ plaques. Curcumin's permeability across the BBB was enhanced by conjugating it with PLGA and lipid‐based carriers, metallic nanoparticles, and targeted agents [124]. Furthermore, curcumin inhibits the formation of Aβ. Aβ aggregates and triggers a pathogenic cascade, with β‐ and γ‐secretases breaking down APP to produce Aβ. Curcumin affected the expression of PS1 and GSK‐3β in cultured neuroblastoma cells. It significantly reduced Aβ40/42 generation, reduced GSK‐3β and PS1 mRNA and protein levels, and raised GSK‐3β protein's inhibitory phosphorylation at Ser9 [49]. Curcumin's therapeutic effects in AD are dependent on its ability to cross the BBB. Nanoparticle technology can enhance curcumin's bioavailability. A stable formulation of curcumin nanoparticles was created using flash nanoprecipitation and freeze‐drying. Tg2576 mice were given either unformulated curcumin, powdered nanocurcumin, or a placebo for 3 months. Nanocurcumin significantly outperformed the placebo in cue memory and improved working memory. Measurements showed nanocurcumin produced a higher plasma concentration and residence duration in the brain [52]. Curcumin has been found to speed up the disintegration of amyloid plaque and inhibit amyloid sedimentation in AD. The research showed the behavioral and molecular effects of curcumin on rat models of AD produced by Aβ (Aβ1‐40‐). It improves spatial memory in AD rats and decreases GFAP‐positive cells and mRNA expression. It also may enhance spatial memory impairments in Aβ1‐40‐induced AD‐induced rats [53]. Innovative curcumin analogues have been developed as potential treatments for AD. It suppresses Aβ aggregation, reduces metal‐induced Aβ aggregation, and chelates metals. It also is beneficial for AD treatment [125]. AD decreases neurogenesis, making targeting endogenous neural stem cells (NSCs) to induce neurogenesis a promising treatment approach. Curcumin‐encapsulated PLGA nanoparticles can stimulate NSC proliferation and differentiation in adult rats, upregulating genes involved in cell proliferation and neuronal development. These nanoparticles restore learning and memory deficits in a rat model of AD‐like symptoms. Curcumin interacts with GSK‐3β, Dkk, and Wif‐1, suggesting it could strengthen brain self‐repair mechanisms and potentially provide a therapeutic strategy for NDs [126]. Curcumin's antioxidant and anti‐inflammatory properties may be due to metal chelation, which can mitigate oxidative neurotoxicity and amyloid formation. Its affinity for copper, zinc, and iron ions was measured using spectrophotometry, showing positive cooperativity with copper and negative cooperativity with iron. It may protect against Aβ toxicity and reduce inflammatory damage [120]. Mn(II) and Fe(III) salts were used to develop curcumin ligand metal complexes. The complexes' ability to reduce Aβ 25‐35 protein accumulation was tested in vivo on Swiss albino mice [127].

### 4.2 Parkinson's Disease

**Figure 4:**
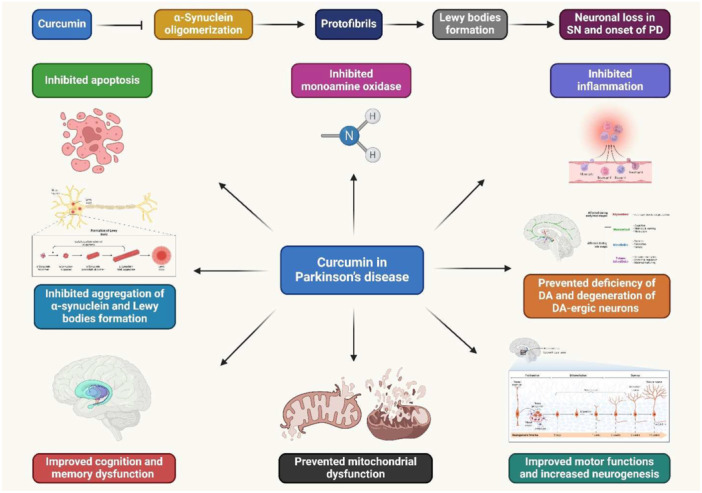
Curcumin showed neuroprotective properties, effectively preventing and treating PD.

PD is an ND caused by a dopamine shortage in the brain and the death of dopaminergic neurons. It causes depression, dyskinesia, and cognitive impairments. Treatment options include dopamine precursors, dopamine metabolism inhibitors, autophagy upregulation, adenosine A2A antagonists, and surgery. Ethnic differences in PD prevalence, particularly in India and Southeastern Asian nations, may be due to dietary variations [128]. Additionally, PD causes a decrease in dopamine in the striatal terminals of dopaminergic neurons, leading to dyskinesia development and inconsistent treatment outcomes. Curcumin has shown potent anti‐inflammatory, antioxidant, and free radical scavenging properties. Its pharmacological activities are mediated by molecular and cellular processes, with α7‐nAChR, a selective α7 nicotinic acetylcholine receptor, being suggested as a potential therapy strategy for PD [129]. Curcumin regulates α‐synuclein aggregation in animal models. A rat model of PD was developed, and curcumin supplementation led to the upregulation of pro‐inflammatory cytokines, NF‐κB, and regulating apoptotic pathway molecules. The study suggests curcumin as a potential molecular targeted therapy for PD (Figure 4) [59]. PD is linked to oligomeric forms of α‐synuclein, which can cause oxidative damage and mitochondrial dysfunction. Curcumin can lower ROS levels, reduce αS‐induced toxicity, and protect cells from apoptosis. This suggests that oligomeric αS, whether extracellularly or intracellularly, may have similar harmful effects [61]. In addition, curcumin inhibits monoamine oxidase B, prevents α‐synuclein aggregation, and counteracts OS [130]. Preclinical research suggests curcumin is a potential neuroprotective drug for PD. A clinical trial assessed the effectiveness of nanomicelle curcumin in improving motor and nonmotor symptoms and quality of life for PD [131]. In addition, curcumin has potential preventive effects on PD in albino rats. It reduces the onset of PD symptoms in a rat model [62]. PD patients experience OS, leading to dopaminergic neuron degradation. Glutathione depletion in presymptomatic neurons leads to OS, mitochondrial malfunction, and cell death. Curcumin protects against peroxynitrite‐mediated mitochondrial dysfunction. Administering curcumin to mice and dopaminergic neurons prevents protein oxidation, releases GSH depletion, and maintains mitochondrial complex I function [60]. Furthermore, curcumin has the potential to treat PD by regulating NDs' PI3k/Akt and BDNF signaling pathways. This neuroprotective drug can prevent the progression of PD by preserving nerve cells [132]. PD is caused by damage to mitochondrial complex I in dopaminergic neurons, likely caused by peroxynitrite. Curcumin pretreatment prevents 3‐nitrotyrosine synthesis and detoxifies brain mitochondria, suggesting potential therapeutic use in NDs as a defense against nitrosative stress [133]. Another research investigated the effect of curcumin on the 6‐OHDA‐PD rat model of hippocampus injury. It increases body weight in rats with 6‐OHDA impairment. It also improves behavioral symptoms and increases monoaminergic neurotransmitters in the hippocampus. It reduces hippocampus damage and increases protein expression, demonstrating its potential for neuroprotection against 6‐OHDA‐induced hippocampal neurons [63]. A study investigates the neuroprotective properties of curcumin in PD experimental models. Curcumin and curcumin‐loaded nanoemulsions (NC) significantly alleviated motor impairment, decreased lipoperoxidation, changed antioxidant defenses, and avoided complex I inhibition. NC was more effective in reducing complex I inhibition and motor impairment compared to curcumin in its free form [64]. Another study demonstrated if curcumin could have a neuroprotective effect on mice's OS, mitochondrial dysfunction, and cognitive impairment caused by rotenone [134]. A study on PD model flies found that exposure to varying doses of curcumin increased their life span, reduced OS and apoptosis, and delayed the loss of activity patterns. It had a strong anti‐PD effect, as it reduced OS and apoptosis, and delayed the loss of activity patterns [135]. Additionally, curcumin can decrease A53T α‐synuclein build‐up by downregulating mTOR/p70S6K signaling and restoring inhibited macroautophagy [65]. Curcumin prevents dopaminergic neurons from dying in a PD mouse model. It inhibits mitochondrial dysfunction by reducing JNK hyperphosphorylation, preventing nigrostriatal degeneration [66]. Furthermore, a study used a mouse model of PD to find the effects of curcumin and its metabolite tetrahydrocurcumin on dopamine and DOPAC depletion [67]. PD is an ND caused by the PINK1 gene mutation, which is essential for maintaining mitochondrial integrity. Curcumin significantly reduced apoptosis and increased MMP and respiration in PINK1 siRNA cells [136]. Curcumin‐glucoside prevents aggregation of α‐synuclein, a key factor in PD. It suppresses fibril and oligomer production under aggregating conditions, suggesting a good stoichiometry for inhibition. Its binding abilities to α‐synuclein monomeric and oligomeric forms were assessed, suggesting it develops disease‐modifying drugs for PD treatment or prevention [137]. A study found that co‐treatment with curcumin‐I reversed these modifications, suggesting a new treatment avenue for curcumin to counteract curcumin‐induced dopaminergic neurotransmission failure, potentially preventing Parkinsonism associated with heavy metals [68]. Another study showed the potential of curcumin as a treatment for mitochondrial dysfunction in PD. The results showed that paraquat interfered with mitochondrial function for all parameters, and curcumin enhanced ATP‐associated respiration. Curcumin's therapeutic use is beneficial when used as a pretreatment before toxin exposure [138]. In addition, curcumin prevents rotenone and salsolinol‐induced damage in PD. This study used SH‐SY5Y cells to simulate dopaminergic neurodegeneration. Results showed synergistic toxicity of modest doses of salsolinol and rotenone, leading to apoptosis and increased caspase‐3 levels. Curcumin pretreatment decreased rotenone and salsolinol‐induced toxicity [139]. Furthermore, curcumin reduces OS and improves locomotor ability in a new PD model. It could be a suitable treatment for PD associated with OS [140]. Curcumin has anti‐inflammatory, iron‐chelating, and free radical‐scavenging properties. It prevents dopaminergic cells from 6‐hydroxydopamine neurotoxicity. This study investigates its neuroprotective effect in PD using a 6‐OHDA‐lesioned rat model [141].

### 4.3 Multiple Sclerosis

MS is a chronic autoimmune inflammatory disease affecting the CNS. Environmental, genetic, and immune factors contribute to its development. Th17 cells, an immune player, induce neuroinflammation, causing demyelination and obstructing nerve signal transmission. B cells also contribute to the disease. OS is a potential cause. Curcumin has neuroprotective effect on MS [142]. Curcumin showed its potential use in treating MS and nerve‐muscle diseases due to its inhibition of pro‐inflammatory cytokine secretion [143]. Conventional medications suppress the autoimmune response, making it a promising treatment for reducing MS neuroimmune imbalance and demyelination. In vitro experiments showed curcumin increased cell proliferation and influenced Th1/Th2/Th17 cytokine production in LPS‐stimulated cells. It also restored motor and behavioral deficits in demyelinated mice [70]. Curcumin neuroprotective effect on MS patients receiving IFN β‐1a treatment. It increases IFN β−1a efficacy [144]. A study investigates the impact of nanocurcumin on inflammatory mediators in relapsing‐remitting MS patients, suggesting that nanocurcumin may reduce neuroinflammation and potentially impact the inflammatory characteristics of MS [145]. Curcumin can inhibit neuroinflammation and alter cell signaling pathways, potentially acting as a neuroprotective and neuropharmacological medication [145]. Additionally, curcumin initiates signaling pathways involved in autophagy, mitophagy, apoptosis, and mitochondrial dysfunction, disrupting MS metabolism [146]. A study found that curcumin nanomicelle significantly increased LH, FSH, testosterone, and TAC concentrations in an MS model [71]. Another study investigates the therapeutic implications and effects of curcumin on the NF‐κB signaling pathway, which is linked to MS [147]. Impairment in RRMS is linked to disruptions in the growth and operation of Treg subpopulations. Nanocurcumin improves MS patients [148]. Moreover, a study found that nanocurcumin can restore dysregulated miRNA expression patterns in MS patients, while some miRNAs were dysregulated in untreated patients. This discovery could suggest that miRNA‐dependent regulation affects B and T cell activity in MS [149]. Curcumin can stimulate astrocytes' anti‐inflammatory properties. It reduced MMP‐9 enzyme activity IL‐6 release, and MCP‐1 mRNA expression. The study found curcumin as a potential therapeutic drug for controlling inflammation‐related disorders caused by astrocytes [150]. In addition, curcumin and calcitriol have immunomodulatory effects. A study on 20 MS patients found that curcumin and calcitriol reduced Th1 and Th17 cell‐related transcription factors and inflammatory cytokines, increased Treg cell frequency, and increased FOXP3 gene expression [151]. Furthermore, curcumin is beneficial for aging and brain pathology due to its anti‐inflammatory, free‐radical‐scavenging, and antioxidant properties [152]. Additionally, curcumin increased glutathione peroxidase‐1 gene expression and antioxidant enzyme activity. The study supports curcumin's efficacy as a treatment agent to reduce the severity of EAE [72]. Another study found that curcumin reduced EAE severity in Lewis rats by reducing inflammatory cells infiltrating the spinal cord. Its dose‐dependently inhibited MBP‐reaction lymphocytes' proliferation and reduced cytokine expression [73]. Curcumin reduces OS in MS. The researchers used Wistar rats immunized against EAE and administered curcumin daily until 24 days after death. The rats showed significant decreases in blood myeloperoxidase enzyme activity and nitric oxide levels, reduced brain malondialdehyde content, and increased serum uric acid levels [75]. EAE is an animal model of MS that causes myelin destruction, leading to increased expression of genes like iNOS and NOGO‐A. Curcumin improved EAE by reducing NOGO‐A expression and enhancing MBP gene expression [153]. Curcumin reduces inflammation and promotes wound healing, improving the MS EAE model [154]. Additionally, curcumin treats immune‐related disorders, including MS. It can modulate the AKT/mTOR autophagy signaling pathway in mice with EAE, a type of MS. This may help balance peripheral and CNS autophagy, potentially preventing neuronal damage and promoting overall health [155]. A study found curcumin's role in CNS demyelination in EAE, it reduces the severity, suppresses IL‐12 production, and inhibits the Janus kinase‐STAT pathway, potentially treating MS and Th1 cell‐mediated inflammatory disorders [77]. Another study found curcumin's potential protective effects against EAE in mice. It reduces EAE severity, potentially decreasing microglia‐mediated inflammation [76]. Reactive CD4+ T cells are essential for MS. Curcumin decreased CD4+ T cell viability and caused TH1, TH17, and Treg cells to undergo apoptosis. It increased active caspase‐3 and Bax expression while decreasing Bcl‐2 expression. It also may prevent effector cell activation and CD4+ T cell growth [156]. Moreover, a study investigated the effects of curcumin on NGF, cortisol, and catalase expression in rats with MS. The rats were divided into three groups: those with MS, healthy control, and curcumin‐treated rats. After 12 days of immunization, curcumin enhanced cortisol levels decreased catalase, and increased NGF expression. The rats' weight also increased significantly after curcumin treatment [157]. In addition, curcumin reduces IL‐12 production in animal models of EAE and MS. It inhibits IL‐12 Rβ1 and β2, IFN‐γ production, and STAT4 phosphorylation while increasing IFNAR subunits 1 and 2, IL‐10 synthesis, and STAT4 phosphorylation [78]. Furthermore, a study using spinal cord homogenate and Freund's adjuvant to generate EAE in mice found that curcumin ameliorated cognitive and motor deficits caused by EAE. It inhibited demyelination of the corpus callosum by activating the AMPK/SIRT1 axis, which activated the CREB/BDNF/MBP pathway. It also reduced OS and neuroinflammation by blocking the JAK2/STAT3 axis, thereby reducing the effects of EAE [158]. Another study demonstrated mitochondrial damage and apoptosis in MS and EAE models using C57 BL/6 EAE mice. Results show that early in EAE, there is mitochondrial damage and oligodendrocyte/neuronal death. Curcumin can prevent apoptosis by blocking the intrinsic apoptotic pathway and protecting mitochondrial damage, preventing overexpression of caspase‐9, caspase‐3, and cyt‐c in EAE mice [159].

### 4.4 Spinal Cord Injury

SCI is a severe disease resulting from direct or secondary injury. Curcumin reduces inflammation by blocking NF‐κB. It improved motor function in rats. The rats also had less gliosis at the contusion site and greater neural element mass. Curcumin therapy could be a new treatment for SCI patients [81]. A study found that curcumin can effectively treat SCI in rats, reducing neurologic impairment, quenching astrocyte activity, and preventing apoptosis and neuron death. Curcumin shows potential for SCI treatment [160]. Curcumin shows potential for treating inflammatory‐based diseases. Research comparing curcumin with conventional treatments like corticosteroids suggests it may more effectively act as the inflammatory source of SCI‐mediated neurological impairment. Curcumin's potential for treating SCI is highlighted, and more preclinical and clinical research is suggested to raise awareness of its potential [161]. Additionally, curcumin's induction of Nrf2 activity reduces inflammatory cytokines, NF‐κB activation, and apoptosis, suggesting it may improve outcomes after SCI [80]. A study found curcumin's impact on spinal cord edema and motor function in a rat model of acute SCI. After injection, curcumin moderately enhanced motor function recovery, lowered bleeding, edema, and neutrophil infiltration, and suppressed the aberrant activation of the JAK/STAT signaling pathway. It is a useful treatment for SCI, potentially inhibiting overexpressed GFAP and AQP4 and activating the JAK/STAT signaling pathway [79]. Another study compared curcumin and methylprednisolone sodium succinate after SCI. Five groups of rats were randomly assigned, and spinal cord tissue samples were assessed. Curcumin treatment improved neurologic outcomes. It reduces SCI consequences by increasing GSH‐Px, SOD, and CAT levels, potentially promoting neuronal survival [82]. After 7 days, the rats receiving curcumin showed higher BBB scores, lower cavity volume, higher SOD activity, lower malondialdehyde activity, and a significant drop in macrophage counts. Curcumin reduces cavitation volume, improves anti‐inflammatory responses, and enhances early functional recovery [162]. A study showed that curcumin reduces inflammation and has protective benefits on SCI. It may improve outcomes after SCI [83]. Another study found that curcumin prevented ischemic SCI in rats. The researchers developed spinal cord ischemia models and administered curcumin intraperitoneally. Results showed an increase in mRNA and protein expression of these receptors, but curcumin significantly reduced their expression, enhancing motor performance and neuroprotection against ischemic SCI [163]. In a study on SCI mice, curcumin significantly suppressed the production of inflammatory mediators and reduced the expression of IκB and IκB kinase. Additionally, it improved functional recovery in mice with impaired hindlimb function after SCI [84]. Curcumin enhances neural stem cell proliferation and aids in functional recovery. It had a modest impact on recovery from severe SCI but helped patients recover more quickly from mild SCI. When combined with stem cell therapy, it led to a dramatic recovery from severe SCI, demonstrating that curcumin regulates stem cell growth in addition to its anti‐inflammatory effects [85]. Another study investigated if curcumin's neuroprotective properties were linked to autophagy regulation. It enhanced spinal cord integrity and reduced neuron apoptosis, suggesting it may enhance autophagy for therapeutic impact on SCI [164]. Curcumin enhanced motor performance after stroke, possibly due to improvements in BSCB integrity. It has significant neuroprotective properties against SCI [165]. Secondary injuries, caused by inflammation, excitotoxicity, calcium overload, and OS, can cause syringomyelia. Curcumin preserves neurons, reduces oxidation and inflammation, and reduces SCI [166]. Additionally, curcumin improved functional recovery and reduced astrogliosis and inflammation in SCI with hyperglycemia [86]. A study showed that curcumin may decrease reactive astrogliosis and lesion cavity activity while increasing SC‐NSPC expression, potentially enhancing functional recovery after SCI in a rat, thereby enhancing the characteristics of SC‐NSPC [167]. Curcumin was administered to male Wistar rats after a SCI, and within the first week, it improved behavioral recovery and reduced NF‐κB activity, reducing the formation of glial scars. It has significant anti‐inflammatory therapeutic potential when administered immediately after the injury [87]. Another study found the role of methylprednisolone (MP) and curcumin in SCI showed that MP had a more effective therapeutic effect than curcumin. The researchers hypothesize that curcumin is given a longer treatment period after SCI. Curcumin may be a more effective treatment option for SCI [168]. Moreover, curcumin can decrease the activation of NF‐κB and transcription‐3 in SCI. In SCI mice, it reduced astrogliosis, NO and IL‐1 expression, and Iba1+ inflammatory cells. It also has therapeutic potential for SCI by promoting healing and preserving residual axons and neurons [169]. SCI can lead to neurological impairments. High‐energy oxidants and free radicals are mediators of secondary SCI, and antioxidants can target these mediators. Curcumin reduces inflammatory damage and repairs SCI by scavenging free radicals and activating Nrf2/HO‐1, making it a viable therapeutic option [170]. Curcumin improves locomotor performance and accelerates neurorepair in a rat model. It significantly decreased inflammatory cytokine expression, alleviated OS, inhibited apoptosis, and improved antioxidant defense mechanisms. It also may protect neurons from ischemia‐reperfusion injury‐related inflammation, OS, and apoptosis [171]. In addition, curcumin alone and in combination with MSC therapy enhanced locomotor ability, facilitated axonal sprouting, and altered regenerative factors and inflammatory responses. Curcumin is a synergistic effect on SCI management [172]. Another study found the potential use of curcumin in treating SCI by modulating the mTOR signaling pathway. It inhibits the activation of this pathway, improving the axon regeneration environment, thus offering a new approach to activate autophagy [173]. Additionally, a study showed the impact of TBI on the hippocampal and spinal cord and the neuroprotective properties of a curcumin derivative. After fluid percussion injury (FPI), rats were given curcumin supplements, which showed protective effects and improved biological activity and brain absorption. This study found that rats with FPI had decreased locomotor performance and learning capacity and that the curcumin derivative restored levels of BDNF and its downstream effectors on neuronal signaling, synaptic plasticity, and OS‐related markers [174]. In addition, a study demonstrated curcumin's impact on motor function and potential neuroprotective role in rats with SCI [175]. Furthermore, another study found the effects of curcumin on inflammatory cytokines and spinal cord labile zinc in rats with traumatic SCI. SCI increased inflammatory cytokines and labile zinc levels in the spinal cord. Curcumin improved apoptosis, spinal cord edema, and hindlimb locomotion impairments. It enhances recovery from SCI by reducing the expression of inflammatory cytokines and labile zinc [176]. Curcumin shows potential in treating SCI. It prevents and treats secondary injuries, improves neurological function recovery, and improves neuronal function after scoliotomy [177]. Combined therapy for SCI using a pH‐responsive polymer‐curcumin conjugate and epSPCs has shown promising results in both acute and chronic cases. The addition of curcumin to a polyacetal improves blood bioavailability, stability, and drug distribution, enhancing neuroprotection and axonal development. This approach could be a promising therapeutic approach for managing persistent SCI [178]. The SRY‐related gene 9 (SOX9) plays a crucial role in glial scar development, influencing the TGF‐β signaling pathway. Curcumin can help in recovery by suppressing NF‐κB and TGF‐β‐SOX9 expression, acting as a neural regeneration mechanism [179]. In addition, curcumin inhibits pro‐inflammatory cytokines, decreases glial fibrillary acidic protein expression, and reduces reactive gliosis. It suppresses TGF‐β1, TGF‐β2, and SOX‐9 production, and reduces chondroitin sulfate proteoglycan accumulation. It also enhances nerve growth conditions [180]. Another study investigated the therapeutic impact of curcumin on reducing bone density below the injury site in rats. After SCI, rats were given curcumin at a dosage of 110 mg/kg body mass per day for 2 weeks. Curcumin prevented bone mass decrease in the tibiae and femurs, maintained bone microstructure, and protected the femoral midshaft. It increased the surface area of osteoblasts and decreased osteoclasts, upregulated osteocalcin mRNA expression, and decreased thiobarbituric acid reactive substances. It also improved the expression of vitamin D receptor (VDR) and Wnt/β‐catenin pathway. These effects may explain curcumin's positive impact on preventing bone loss in rats [181]. Curcumin treats neuroinflammatory and NDs, including posttraumatic inflammation after acute SCI. This study investigated the effects of curcumin on astrocyte reactivation, specifically injury‐induced RANTES. Curcumin inhibited the effects of injury‐induced RANTES and increased lipid peroxidation. It reduced RANTES expression in reactivated astrocytes, suggesting its potential use in treating SCI by reducing robust RANTES generation [182]. Furthermore, curcumin treatment reduces inflammatory cytokine expression and labile zinc accumulation in rats with traumatic SCI, potentially enhancing recovery from the injury [176].

### 4.5 Stroke

Stroke is a cerebrovascular disease. Curcumin is a potential treatment for ischemic stroke by altering the microglial phenotype from pro‐inflammatory to anti‐inflammatory and tissue‐reparative. Curcumin post‐treatment decreased cerebral ischemia damage, improved sensorimotor abilities, and decreased M1 polarization produced by lipopolysaccharide and interferon‐γ. Curcumin also decreased pro‐inflammatory cytokines without affecting the survival of microglial cells [89]. A study found potential epigenetic effects of curcumin during stroke and molecular modifications that may improve its bioavailability. It modulates gene expression [183]. Curcumin protects the body from ischemia and hemorrhagic stroke, with beneficial effects even at low concentrations when delivered through nanostructured devices. It has antiapoptotic, antioxidant, and neuroprotective properties that help repair damaged brain tissue and preserve the BBB's integrity [184]. Another study found curcumin pretreatment on BBB integrity during acute ischemic stroke. It enhanced neurological scores, reduced infarct size, preserved synaptic remodeling, and increased tight junction protein expression. It also reduced inflammation mediators MMP‐9 and NF‐κB. It may reduce brain damage [90]. Additionally, research found the impact of curcumin on Notch signaling in rats, which could promote neurogenesis and prevent focal cerebral ischemia‐reperfusion damage. After the stroke, rats treated with curcumin showed fewer neurobehavioral impairments, higher NICD levels, and more BrdU/DCX‐positive cells than those treated with a vehicle. Curcumin may activate the Notch signaling system [91]. In a study using Wistar rats, curcumin‐treated MCAO showed downregulated markers associated with apoptosis and inflammation, indicating its potential as a useful adjunctive agent in preventing cerebral I/R injury [92]. In addition, research found curcumin in treating stroke in an animal model. The rats were infected with MCAO and reperfusion, and their brain levels were measured. It significantly decreased cerebral water volume and edema and decreased mitochondrial membrane potential. It is used to treat stroke [185]. The researchers used a rat model and sirtinol, an inhibitor of Sirt1, to investigate the underlying process. It had a neuroprotective effect, improving neurological scores and decreasing brain edema and infarct volumes. It also had anti‐inflammatory effects, increasing mitochondrial cytochrome c levels and complex I activity. Sirtinol reversed the effects of curcumin, suggesting that its therapy decreases ischemic stroke‐induced brain damage [186]. Furthermore, a study found the potential of curcumin to protect against strokes. Stroke‐prone rats were given either saline or curcumin daily. It significantly delayed stroke onset and improved survival. This was due to reduced ROS and improved carotid artery relaxation. Curcumin, when inhibited by Genipin, prevented the rise in NO generation or reduction in ROS [187]. Additionally, another study found the impact of curcumin on neuronal apoptosis in mouse N2a cells following oxygen‐glucose deprivation/reoxygenation (OGD/R) and cerebral ischemia/reperfusion (I/R) injury. Results show that curcumin treatment decreases infarct magnitude, enhances neurological function, reduces neuronal damage, and increases neuronal survival rate. Curcumin enhances cell survival in mouse N2a cells. It prevents ischemia‐induced mitochondrial apoptosis by limiting Bax activation, potentially highlighting its neuroprotective benefits [93]. Curcumin regulates the NLRP3 signaling pathway in ischemic brain injury. It has neuroprotective properties, preventing neuronal death and reducing inflammation. The study also discusses the function of medications targeting NLRP3 and the potential for curcumin therapy to cross the BBB. It could potentially delay ischemic brain injury [188]. Another study suggested curcumin is a potential treatment for stroke by inhibiting microglial pyroptosis, a pro‐inflammatory pathway influenced by stroke. It improved sensorimotor performance in mice post‐60‐min middle cerebral artery occlusion. It also inhibited the NF‐κB pathway, enhancing sensorimotor function and reducing white matter damage [94]. Curcumin treats nerve tissue. A study found that a 30 mg/kg dose of curcumin nanoemulsion reduced weight loss, reduced hemorrhage, and improved behavioral recovery in Wistar rats. The nanoemulsion also regulated antioxidant responses, suggesting potential therapeutic benefits against ICH [95]. A study investigates the pathophysiology of visual impairment in stroke. Results showed that rats with hypertension had more severe intraocular pressure (I/R)‐induced retinopathy. Curcumin treatment significantly decreased apoptosis in retinal capillary cells and neurons [189]. In addition, another study investigated the forebrain's ability to survive ischemia and reperfusion in rats given a long‐term curcumin dose. Results showed higher enzyme activities in the forebrain tissue in group I, while enzyme activities in other groups were lower. The neurological score of curcumin‐treated groups was lower than the ischemia group, and the apoptotic index decreased in curcumin‐treated groups. Curcumin may be neuroprotective after ischemia and reperfusion injury [96]. Curcumin treatment increased Prdx6‐positive neuronal cells and protein expression, reduced OS, and produced neuroprotective benefits against ischemia damage [97]. Furthermore, a study found the potential of combining curcumin and human umbilical cord‐derived mesenchymal stem cells (hUC‐MSC) for treating acute ischemic stroke. Results show that curcumin‐hUC‐MSC therapy improves neurological function and anti‐inflammatory microglia polarization, enhancing the therapeutic efficacy of hUC‐MSC transplantation [98]. Another study found that curcumin, when combined with conventional stroke therapy, significantly improved patients' muscle power and quality of life. This study found that patients receiving curcumin adjuvant treatment showed improvements in energy, mobility, thinking, extremity function, and work productivity. Additionally, there was a significant decrease in stroke‐related blood pressure after 3 months of treatment [190]. Curcumin‐piperine supplementation improved various blood pressure parameters in ischemic stroke patients during rehabilitation, reducing their mortality rate and social and economic costs [191].

### 4.6 Amyotrophic Lateral Sclerosis

ALS is a disease‐causing motor neuron loss [192]. ALS is a motor neuron disease that is characterized by the loss of top motor neurons in the cerebral cortex and lower motor neurons in the spinal cord. The pathogenic causes of ALS are complex and include OS, which causes mitochondrial dysfunction and motor neuron loss. Anti‐inflammatory and antioxidative medicines are potential treatments [193]. Curcumin regulates diminished SOD1, one of the primary proteins linked to ALS, during its early aggregation phases [194]. In addition, curcumin is used to treat ALS, a disease protein linked to DNA damage, transcriptome alterations, and altered cellular stress response [195]. ALS affects motor neurons. Abnormal SOD1 deposition accounts for 20% of ALS cases. Curcumin suppresses SOD1 fibrillation and promotes smaller, disordered aggregates. It reduces toxicity by attaching itself to amyloidogenic areas and preventing toxic species development. Curcumin bulk and enhanced aqueous solubility nanoparticles show comparable aggregation control, suggesting potential use in ALS management [196]. A study involving patients with ALS found that showed an oral curcumin supplement reduces disease development, improves aerobic metabolism, and reduces oxidative damage. The study found that Group B showed a consistent score on the ALS‐FRS‐r, decreased AOPPs, and maintained constant FRAP exercise values. Curcumin treatment also reduced exercise AOPPs, similar to controls. This study suggested a minor reduction in disease development and improved understanding of ALS pathogenic pathways [197]. Another study investigated the potential therapeutic benefits of curcumin derivative GT863 for ALS. GT863 inhibited SOD1 aggregation, preventing oligomeric aggregation. GT863 reduced motor dysfunction progression and preserved big neurons in the spinal cord [99]. Curcumin has antioxidant defense mechanisms increase and mitochondrial dysfunction is reduced, potentially aiding in the treatment of ALS. The study found the importance of curcumin in reducing oxidative damage and promoting antioxidant defense mechanisms in ALS pathophysiology [198]. Oral curcumin may help treat PALS through four processes. Three PALS experienced motor improvements, one pilot trial showed some benefit, and defective preclinical studies showed benefits [199]. Another study found that human TDP‐43, a key protein in ALS pathological inclusions, causes mitochondrial abnormalities, decreased activity, and increased expression of UCP2. Dimethoxy curcumin could potentially improve mitochondrial dysfunction in mutant TDP‐43 cells, potentially aiding in treating NDs associated with mutant TDP‐43 [100]. Furthermore, a study investigated the aggregation effect of native and mutant SOD1 in ALS. Curcumin inhibits SOD1 aggregation. To develop ALS treatments, this study suggested a traditional method for treating mutant SOD1 using curcumin [200].

### 4.7 Huntington's Disease

HD is an ND caused by an abnormal increase in the polyglutamine repeat in the Huntingtin protein. PolyQ's pathophysiology is complex, requiring multi‐targeted treatments. Curcumin is a promising candidate for minimal side effects in polyQ treatment, as it improves neuronal function, neurodegeneration, and cytotoxicity. Curcumin inhibits cell death in a Drosophila model, potentially slowing HD progression [201]. A study investigated the therapeutic potential of curcumin‐supplemented diets on peripheral and cerebral dysfunctions in R6/2 mice, a well‐studied HD animal model that mimics human disease [101]. Curcumin has significant therapeutic properties due to its ability to modify molecular pathways and protect neurons from degeneration in HD. It treats or prevents HD, potentially inhibiting disease progression and showing the way for more efficient treatment agents [202]. Additionally, curcumin‐encapsulated solid lipid nanoparticles (C‐SLNs) show potential in treating HD, activating the nuclear factor‐erythroid 2 antioxidant pathway, and improving neuromotor coordination [102]. In addition, curcumin has been investigated for its potential to facilitate early pathogenic phenotype in mice. Curcumin‐fed KI mice showed reduced huntingtin aggregates, elevated striatal DARPP‐32 and D1 receptor mRNAs, and ameliorated rearing impairments. This study found a net positive effect of curcumin in HD, including improvements in transgene‐dependent measures [103]. Another study showed HD affects circadian timing and evaluated the effectiveness of melatonin and curcumin in stopping motor deficits. They measured mRNA expression of transcriptional feedback loop genes and found that both curcumin and melatonin restored the 24‐h rhythm in Period and Timeless mRNA expression, enhancing HD and exhibiting eclosion behavior. Curcumin and melatonin may be useful therapeutic drugs for HD treatment [104]. A study investigated the effects of solid lipid curcumin particles (SLCPs) and solid lipid particles (SLPs) on HD in 11‐month‐old mice. Both treatments improved learning and memory performance and maintained MSN arborization, spinal density, and synaptic protein levels. The research suggests that both lipid particles and SLCPs can have therapeutic effects on the recovery of HD brain pathology and cognitive deficits in elderly YAC128 HD mice [203]. Curcumin effectively reduced symptoms in HD flies. It controlled body weight, lipid content, and carbohydrate levels, reduced ROS, and improved flies' survival abilities. It also reduced ROS levels, improved locomotor function, and enhanced survival in advanced disease stages. It effectively reduces metabolic disturbances in HD flies and may improve HD‐related complications [204]. Another study demonstrated curcumin's potential for immunomodulation in HD. It significantly decreased the number of higher crystal cells and phenoloxidase activity in sick flies. It also increased the phagocytic activity of plasmatocytes, reduced ROS levels, and mitochondrial dysfunction in sick flies. Additionally, it reduced the transcriptional expression of AMPs and pro‐inflammatory cytokines. It can effectively reduce immunological abnormalities in HD flies and may ease HD‐related complications [105]. Curcumin therapy promotes mutant huntingtin aggregation and cell death, while antioxidant N‐acetyl cysteine reverses this dysfunction and prevents cell death [205]. Furthermore, curcumin is a treatment strategy for HD. Researchers have developed biodegradable nanoparticles containing curcumin, which improve its transport and brain bioavailability. Curcumin‐encapsulated nanoparticles can infiltrate cells in an in vitro model of HD, thereby decreasing their sensitivity to apoptosis [206].

## 5 Preclinical and Clinical Studies

Preclinical research shows curcumin can treat various types of cognitive impairment and the mechanisms leading to age‐related cognitive decline. Clinical trials are still conflicting, but curcumin's potential is strong [207]. Preclinical models investigated curcumin's potential role in anxiety, bipolar disorder, PTSD, and autistic spectrum disorders, despite the lack of evidence. The study showed curcumin's cellular targets and its role in psychiatric diseases like anxiety and depression, as well as its impact in preclinical and clinical trials [208]. A study found the benefits of curcumin and its formulations in preclinical and clinical studies of neuropathic and postoperative pain. Curcumin potentially prevents or reduces neuropathic and postoperative pain disorders, according to promising preclinical and clinical trials. Clinical research is needed to explore the use of curcumin as a supplement to neuropathic and postoperative medications [209]. Curcumin's effectiveness in reducing depression symptoms has been demonstrated through numerous preclinical studies and numerous clinical trials [210]. Another study showed curcumin's potential protective effects on animal models of diabetic neuropathy, tardive dyskinesia, and severe depression, promoting clinical trials to explore its neurological applications [211]. Preclinical and clinical research on the management of pathological pain is found in this study. Clinical research is needed to assess the clinical efficacy of curcumin in treating pathological pain. Further research is required to determine possible strategies for improving curcumin's bioavailability and water solubility [212]. Due to recent failures in late‐stage AD clinical trials and preclinical indications of AD decades before clinical manifestation, intervention aims to postpone or prevent AD onset. Longitudinal curcumin intervention studies should include older, healthy community residents and those with subjective memory issues in long‐term, longitudinal follow‐ups. The clinical significance of curcumin in preventing AD and cognitive loss (Table 2) is expected to increase with the addition of neuroimaging and AD‐related biomarkers [115]. Preclinical research indicates curcumin improves cognition in AD and nonpathological aging. However, few human studies consistently show curcumin may stabilize cognitive decline. Curcumin's broad availability and safer profile make it a promising substitute for existing therapies. Its potential to prevent cognitive deterioration and its potential as a natural substance makes it a promising alternative [213]. A study is a preliminary investigation into the impact of curcumin on clinical scores and misfolded p‐syn in PD patients. Curcumin supplementation significantly reduced the worsening of clinical parameters and p‐syn load, suggesting curcumin's potential to reduce skin p‐syn accumulation in PD patients [214]. Inadequate target design and preclinical models can lead to low translation rates in clinical trials. Improved animal models are needed to mimic human pathophysiology and forecast results accurately. Clinical trials face challenges in selecting the best target population, patient stratification, accurate clinical diagnosis, and distinguishing between disease‐modifying effects and symptomatic effects. Delayed‐start trial designs have been used to address these issues. Factors like the placebo effect, intersite variances, insensitivity of rating scales, rater bias, and trial lengths can lead to uncertain impacts in mild to moderate patients, limiting the effectiveness of neuroprotective drugs. Additionally, determining the best dosing regimens for adequate brain penetration is a challenge, requiring extensive preclinical and early clinical research, developing subtherapeutic doses, and evaluating dose–response correlations. The development of effective neuroprotective medicines faces numerous theoretical and methodological constraints, inhibiting their clinically significant results at every stage of the research process. New techniques in network pharmacology, drug repositioning, multitarget approaches, and clinical research could lead to innovative solutions. This study found neuroprotective drugs currently being studied in clinical phases for treating ALS, AD, PD, and HD [215]. Clinical research indicates potential for novel therapies, including riluzole and edaravone for ALS and fingolimod, dimethyl fumarate, and IFN β−1b for MS, with turmeric showing improvement in patients [216].

**Table 2:** It discusses the preclinical and clinical studies of curcumin.

Duration	Species/disorder age (*N*)	Study design, dose, and route of administration	Cognitive measurements	Major finding	References
Three weeks	Male Wistar rats (*N* = 40; *n* = 10/group)	(1) Sham:1operation + vehicle; (2) sham2: operation + curcumin (80 mg/kg, p.o); (3) STZ + vehicle, p.o; (4) STZ + curcumin (80 mg/kg, p.o)	MWM, PA	Reversed the alteration in OS markers and cognitive and behavioral traits induced by ICV‐STZ	[217]
Three weeks	D‐galactose‐injected SC (100 mg/kg) C57Bl/6 male mice—10 weeks *n* = 10	300 mg/kg gavage for 3 weeks w/ (Curcumin‐DGAL) or w/o D‐galactose (curcumin)	MWM	Enhanced Ki67 immunoreactive nuclei in the dentate gyrus's subgranular zone	[218]
Thirty days	Male Wistar rats (*N* = 35; n1 = 7, n2 = 7, n3 = 6, n4 = 8, n5 = 7)	(1) Sham group; (2) STZ infused group; (3) STZ + curcumin (25 mg/kg, p.o); (4) STZ + curcumin (50 mg/kg, p.o); (5) STZ + curcumin (100 mg/kg, p.o)	OFT, OLT, ORT, EPM, Y‐Maze	Inhibited short‐term recognition but not spatial memory at high dosages	[219]
Four months	Male and female C57Bl/6J mice (N = 24; n1 = 9, n2 = 7, n3 = 8)	(1) Wild‐type animals; (2) control hTau mice; (3) hTau + curcumin‐Longvida (500 ppm in chow)	MWM, Y‐Maze, NORT	Improved cognitive performance in mice and decreased the formation of soluble tau aggregates	[220]
Eighteen weeks	Male and female C57BL/6 mice (*N* = 30; *n* = 10/group)	(1) WT group; (2) 5XFAD, control; (3) 5XFAD + 5 mg/kg curcumin, i.n	Y‐maze	Protected memory deficits and decreased the formation of Aβ plaques	[221]
Eighteen months	Nondemented 51–84 years (*N* = 40; n1 = 19, n2 = 21)	(1) Placebo; (2) Theracurmin (90 mg curcumin) orally, b.i.d	(1) SRT; (2) BVMT‐R; (3) trail making test	Enhanced memory and attention performance and protected neuropathological deposition in the amygdala and hypothalamus	[222]
Four weeks	Healthy elderly 60–85 y (*N* = 60; n1 = 30, n2 = 30)	(1) Placebo; (2) 400 mg Longvida (assessment: 1 and 3 h and 4‐week treatment)	(1) COMPASS; (2) DASS21; (3) CFS; (4) BL‐VAS; (5) STAI	Enhanced attention and working memory performance, improved work memory after 4 weeks	[223]
Six months	Cognitive decline/possible AD > 50 (*N* = 27; n1 = 8, n2 = 8, n3 = 11)	(1) Placebo; (2) curcumin (1 g); (3) curcumin (4 g)→curcumin was given either as a powder or in capsules	MMSE	The bioavailability of 1–4 g of curcumin is comparable, but it is higher when taken in capsule form	[224]

## 6 Toxicology Studies

Aluminum, a neurotoxic substance, is linked to AD due to its neurotoxic properties. Prolonged aluminum exposure increases Aβ levels and oxidative damage. Current treatments only improve symptoms, making new medications with fewer adverse effects necessary. A study showed that long‐term curcumin treatment prevents rats from oxidative damage and cognitive impairment caused by aluminum. The rats were given 100 mg/kg of aluminum chloride intraperitoneally and concurrently with curcumin. Chronic curcumin administration reduced oxidative damage and improved memory retention [225]. Another study found curcumin's potential as a treatment for lead‐induced neurotoxicity in rats. This study involved 36 male Sprague Dawley rats divided into five groups: recovery, lead‐treated, treatment group 1, and treatment group 2. After receiving the lead, the rats' motor scores and SOD activity decreased, while their malondialdehyde levels increased. However, curcumin treatment enhanced motor performance, decreased lead in the cerebellum, improved OS markers, and repaired the cerebellum's histological structure. Curcumin's therapeutic potential and broad pharmacological safety border suggest it could be developed as a natural medication to treat lead toxicity [226]. In addition, a study demonstrated that curcumin acts as an antidote for toxic agent‐induced neurotoxicity. It has antidotal actions that suppress OS, inflammation, and apoptosis. It also protects against harmful substances in animal organs, including the brain [227]. A study investigated curcumin analogues' BBB permeability and potential to protect against Aβ toxicity in Caenorhabditis worms [228]. Additionally, another study showed the impact of curcumin on Aβ40 aggregate formation and neurotoxicity caused by Aβ40 aggregates. It enhances the population of harmless prefibrillar aggregates and “off‐pathway” soluble oligomers, reducing toxicities caused by various Aβ conformers. It also decreased cell membrane permeabilization, suggesting a potentially membrane‐mediated neuroprotective action. It protects against Aβ‐induced toxicity through two coordinated mechanisms [229]. Furthermore, this study demonstrates curcumin's antagonistic action against arsenic trioxide (ATO)‐induced neurotoxicity in ducks. The ducks were divided into four groups and exposed to different concentrations of ATO. ATO exposure inhibited duck growth and increased the rate of arsenic accumulation. Curcumin also reduced the expression of pro‐inflammatory and anti‐oxidation proteins. ATO treatment may lead to BBB damage [230]. Curcumin protects neuronal cells from Aβ‐induced damage by increasing antioxidant enzyme levels and reducing OS, DNA damage, intracellular calcium influx, and tau hyperphosphorylation in PC12 cells [231]. Moreover, a study found curcumin's impact on AD neuronal toxicity. It significantly reduced iAβ‐induced toxicity in primary cultured rat prefrontal cortical neurons. It prevented iAβ‐induced decreases in p‐AKT contents. It is suggested that may protect neurons from iAβ‐induced cytotoxicity [232]. In addition, another study found that curcumin significantly protects primary prefrontal cortical neurons from Aβ‐induced toxicity in cultured rats. It increases caspase‐3 content and reduces Bcl2 content, inhibiting Aβ25‐35‐induced increases in caspase‐3 and Bcl2 content. These protective effects are mediated by both Bcl2 and caspase‐3 [233]. Curcumin decreases the toxicity of α‐Syn aggregates in PD by altering their hydrophobic surface exposure [234]. Furthermore, curcumin protects against arsenic‐induced neurobehavioral toxicity in rats by regulating OS and dopaminergic functions [235]. Additionally, curcumin inhibited OS and mitochondrial cell death pathways, suggesting potential neuroprotective properties for A53T α‐synuclein‐linked Parkinsonism and other genetic Parkinsonism forms, preventing toxicity in a PD PC12 inducible cell model [236]. Curcumin protects rat hippocampus neurons from STS‐induced cytotoxicity [237]. A study evaluated the potential aluminum exposure in rat brain synaptosomes and the therapeutic and protective effects of curcumin on biochemical and morphological changes. Results showed increased OS, apoptosis, and ultrastructural changes. Curcumin showed a greater degree of therapeutic than protective effects in AlCI3‐induced neurotoxicity [238]. Another study evaluated curcumin for potential antioxidant effects on the neurotoxicity of Acrylamide in rats. Results showed curcumin preserves TERT‐related antiapoptotic function, potentially protecting brains intoxicated with Acrylamide [239]. Furthermore, curcumin can pass through the BBB, potentially treating NDs linked to inflammation, OS, and apoptosis. It reduces neurotoxicity in a specific animal model of AD [55].

## 7 Conclusion and Future Perspectives

Curcumin has emerged as a promising neuroprotective drug in the fight against NDs such as AD, PD, HD, MS, ALS, SCI, and stroke. It changes cellular and molecular signaling pathways to effectively target Aβ aggregation, OS, and neuroinflammation, which are three major processes that damage neurons. Curcumin primarily enhances antioxidant defenses and reduces inflammatory responses by activating the Nrf2 pathway and suppressing NF‐κB, which contributes to its neuroprotective properties. It improves neurons' health and function by promoting autophagy and neurotrophic factor production. Clinical research indicates that curcumin's potential for therapeutic use is due to its ability to penetrate BBB capabilities. It has anti‐inflammatory properties and low toxicity, making it an effective long‐term treatment option for NDs, potentially modifying their progression and improving patient outcomes. Despite curcumin has medicinal potential, resolving a few issues is necessary to effectively utilize its neuroprotective advantages. The primary focus is on improving curcumin's bioavailability to improve its clinical effectiveness due to its limitations in absorption and metabolism. Future research should explore the precise molecular mechanisms of curcumin's effect to better understand its interaction with various signaling pathways. Additionally, investigating the synergistic effects of curcumin with other medicinal agents could enhance its efficacy and expand its use in combination therapies. It will be beneficial for neuroprotective benefits in other NDs. Curcumin's clinical translation is inhibited by its quick metabolism, low BBB permeability, and poor bioavailability, despite its strong neuroprotective potential. Nanotechnological strategies, such as liposomal formulations, structural analogs, and nanoparticle‐based drug delivery systems, are being explored to enhance curcumin's pharmacokinetics and target specificity. Future studies should concentrate on developing curcumin formulations that are optimized for solubility and sustained release to ensure effective brain penetration. Furthermore, clinical trials are required to assess curcumin's efficacy, optimal dosage schedule, and long‐term safety profile in individuals with ND. Combining curcumin‐based treatment with traditional pharmaceutical therapies and customized medicine techniques may offer potential synergistic advantages. Curcumin's potential as a natural neuroprotective drug requires addressing pharmacokinetic issues and conducting clinical trials, requiring a multidisciplinary approach involving nanotechnology, neuropharmacology, and systems biology.

## Data Availability

The data that support the findings of this study are available from the corresponding author upon reasonable request.
